# Bioactivity Guided Fractionation of Leaves Extract of *Nyctanthes arbor tristis* (Harshringar) against *P falciparum*

**DOI:** 10.1371/journal.pone.0051714

**Published:** 2012-12-26

**Authors:** Pinky Kumari, Dinkar Sahal, S. K. Jain, Virander S. Chauhan

**Affiliations:** 1 Malaria Research Laboratory, International Centre for Genetic Engineering and Biotechnology, Aruna Asaf Ali Marg, New Delhi, India; 2 Department of Biotechnology, Jamia Hamdard, Hamdard Nagar, New Delhi, India; State University of Campinas, Brazil

## Abstract

**Background:**

*Nyctanthes arbor-tristis* (Harshringar, Night Jasmine) has been traditionally used in Ayurveda, Unani and other systems of medicine in India. The juice of its leaves has been used by various tribal populations of India in treatment of fevers resembling malaria.

**Aim of the study:**

This work reports the antiplasmodial activity guided fractionation of Harshringar leaves extract.

**Methodology:**

Crude ethanolic Harshringar leaves extract and its RPHPLC purified fractions were studied for antiplasmodial potency against 3D7 (CQ sensitive) and Dd2 (CQ resistant) strains of *P.falciparum* and subsequently subjected to bioassay guided fractionation using reverse phase chromatography to pursue the isolation of active fractions.

**Principal Findings:**

Harshringar crude leaves extract and some of its RPHPLC purified fractions exhibited promising antiplasmodial potency against 3D7 and Dd2 strains of *P.falciparum*.

**Conclusions:**

The present study has provided scientific validity to the traditional use of leaves extract of Harshringar against malaria leading to the conclusion that this plant holds promise with respect to antimalarial phytotherapy. This is the first scientific report of antiplasmodial activity of RPHPLC fractions of Harshringar leaves extract against *P.falciparum* strains.

## Introduction

Malaria has been reported to be endemic in 106 countries, infecting 216 million and killing 655000 persons in 2010, mostly pregnant women and children under five. Among five species of *Plasmodium* that affect humans, malaria due to *P. falciparum* is the most deadly [1]. In spite of several control programmes, there has been very little improvement in control of malaria which leads to both economic and human losses. Wide spread resistance to common antimalarial drugs has worsened the problem [2]. Furthermore, no new class of antimalarials has been introduced into clinical practice since 1996 [3]. Consequently new drugs/drug combinations are urgently needed today for malaria, which should have novel mode of action/be chemically different from the drugs in use currently [4].

Natural products, particularly medicinal plants, have been important sources of new drugs, new drug leads, and new chemical entities throughout the history and continue to serve as the basis for many pharmaceuticals used today [5]. It is estimated that 80% of people in developing countries still use plant based traditional medicines [6]. The therapeutic success of two important plant derived compounds quinine and artemisinin against malaria has inspired researchers to search new antimalarials from plants.

India, with its wealth and variety of medicinal plants has accumulated over generations a great mass of popular remedies. *Nyctanthes arbor tristis* (Family: Oleaceae), commonly known as Harshringar (English- Night Jasmine) is one of them [7]. *Nyctanthes arbor-tristis* is a large shrub with fragrant flowers found wild in forests of central Indian, sub-Himalayan regions and commonly cultivated in gardens in many parts of India [8]. The leaves are opposite, simple, 6–12 cm long, 2–6.5 cm broad, with an entire margin having good medicinal properties [9], [7]. The decoction of leaves is extensively used by Ayurvedic physicians for treatment of arthritis, obstinate sciatica, malaria, intestinal worms and as a tonic, cholagogue and laxative [10]–[13]. This plant has also been found to possess leishmanicidal [14], [15], amoebicidal [16], anti helminthic [17], antipyretic potency [18]. Scientific literature on the antimalarial properties of Harshringar leaves has remained restricted to investigations on crude extracts alone [19]–[21]. In the present study, bioactivity guided Reverse Phase High Performance Liquid Chromatographic (RPHPLC) fractions of Harshringar leaves extract have been evaluated against Chloroquine (CQ)-resistant (Dd2) and CQ-sensitive (3D7) strains of *P.falciparum*.

### Ethics statement

Although our centre (ICGEB, New Delhi, India) has an ethics committee {Institutional Animal Ethics Committee (IAEC)}, we did not seek the approval of this committee for the experiments reported in our manuscript. Such an approval was not felt necessary since our study does not have any human subjects. We are associated with the a registered blood bank (Rotary Blood Bank, Tughlakbad Institutional Area) at New Delhi, India for supply of human red blood cells used in culture of *P.falciparum*.

## Methods

### Collection and identification of Harshringar leave

Harshringar leaves were collected from International Centre for Genetic Engineering and Biotechnology (ICGEB) campus, New Delhi, India. The leaves were identified, authenticated in Raw Materials Herbarium and Museum (RHMD), NISCAIR, India by Dr. H. B. Singh (Voucher specimen No. 2043). The voucher specimen was deposited in RHMD for further reference.

### Preparation of aqueous and ethanolic extracts

After washing, draining of water, leaves were air-dried in shade at room temperature (25±5°C) for 7–10 days on laboratory bench, stalks removed and the dried leaves were powdered with hammer mill to yield 840 grams (g) of powder. Eight hundred forty grams of powdered leaves were extracted with 5.65 liters of milli-Q water in 10 liter plastic beaker under agitation on shaker at room temperature for 19 hrs. The extract was filtered with help of muslin cloth. ∼50 mL of filtered water extract was lyophilized to a dry powder (CWE). Residual leaves so obtained were air-dried in shade and subjected to soxhlet ethanol extraction.

827 g of residual leaves were consecutively extracted with ethanol (1 litre×6) in a Soxhlet apparatus at 65°C. The crude ethanol extract (CEE) was evaporated to dryness under reduced pressure on rotary evaporator (Rotavapor, Buchi) at 37°C and further dried in vacuum dessicator under phosphorus pentoxide (P_2_O_5_). The dark brown sticky extract {2.1% (∼17 g)} was stored at 4°C for further use. CWE and CEE were assayed for antiplasmodial potency against *P.falciparum* 3D7 using CQ as positive control.

### Reverse phase chromatographic fractionation of CEE

The active CEE was fractionated by reverse phase chromatography on a glass column. Briefly 6.5 grams of CEE (dissolved in methanol) and 6 grams of coarse Bondapak C_18_ resin [{37–55 µm (micro meter), 125 Å (Angstrom)}, soaked in Methanol] were mixed together. The excess methanol was evaporated on rotary evaporator at 37°C. The sample adsorbed resin was packed in glass column. First dimension reverse phase chromatographic elution was done by step gradient, starting with milli-Q water (220 mL), followed by Methanol-water volume/volume (v/v): 20% (200 mL), 40% (200 mL), 60% (300 mL), 80% (250 mL), 100% (250 mL), acetonitrile-water v/v: 50% (200 mL), 75% (200 mL) and isopropanol (200 mL) as eluent. Eluates were concentrated to dryness under reduced pressure on rotary evaporator at 37°C, further dried in vacuum dessicator under P_2_O_5_. This fractionation resulted in nine fractions {Water Fraction, 20% Methanol Fraction (MF), 40% MF, 60% MF, 80% MF, 100% MF, 50% Acetonitrile Fraction (ACNF), 75% ACNF, and Isopropanol Fraction}. Each fraction was weighed and stored at 4°C for further use. All nine fractions were tested for antiplasmodial potency against *P.falciparum* 3D7. The two most active fractions namely 60% MF (150 mg) and 80% MF (300 mg) were pooled together, dissolved in dimethyl sulfoxide (DMSO) (15 mL) and subjected to bioassay guided 2^nd^ dimension RPHPLC.

Analytical reverse phase chromatographic separations were performed on analytical C_18_ column (Deltapak, C_18_, 300×7.8 mm, 15 µ, 300 Å) using methanol-water, linear gradient 20–95% at a flow rate of 2 mL/min over 48 minutes. Dual wavelength detector was set at 214/280 nm. An aliquot of 200 µL (Stock- 3.75 mg/mL DMSO, Amount injected ∼750 µg) was injected for analytical RPHPLC. An aliquot of 1 mL (Stock-90 mg/1.5 mL DMSO, Amount injected ∼60 mg) was injected on semi-prep reverse phase C_18_ column (Waters Deltapak, C_18_, 300×19 mm, 15 µ, 100 Å) using methanol-water linear gradient 20–95% at a flow rate of 10 mL/min over 90 minutes. Fifty five fractions (14 mL each) were collected using automated fraction collector. These 55 fractions were judiciously pooled together to total of 10 pools (P1–P10), each of which was tested for antiplasmodial potency against *P.falciparum* 3D7.

**Figure 1:**
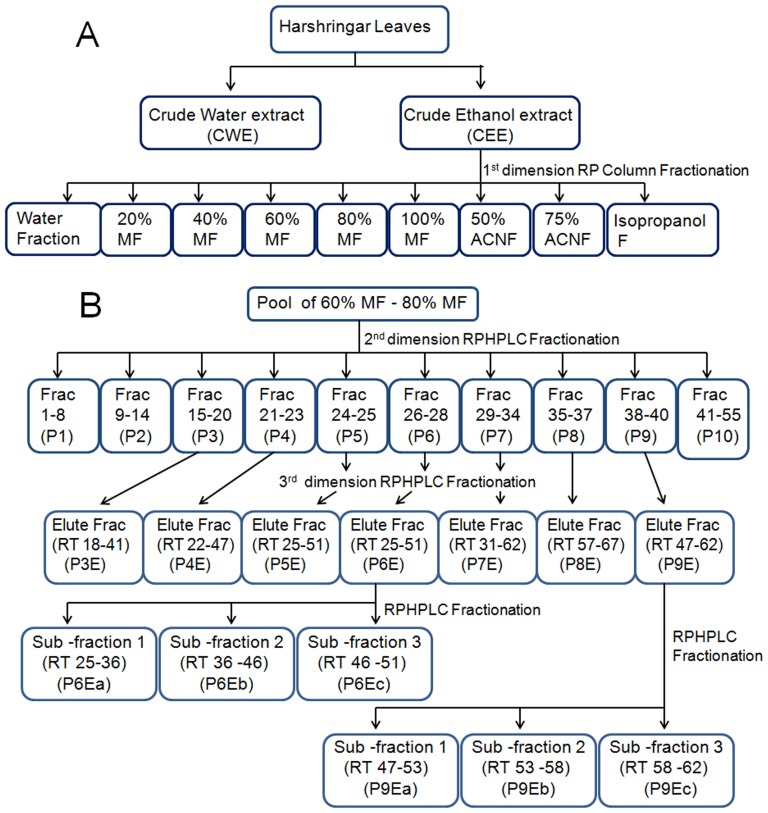
Scheme of extraction and reverse-phase fractionation of *Nyctanthes arbor-tristis* (Harshringar) leaves. Panel A shows scheme of solvent extractions and 1^st^ dimension reverse phase (C_18_) fractionation of ethanolic extract of leaves. MF (Methanol Fraction), ACNF (Acetonotrile Fraction). Panel B shows 2^nd^ and 3^rd^ dimension RPHPLC fractionation of 60% –80% MF. RT (Retention time in minutes).

Pools (P3–P9) were further purified by 3^rd^ dimension RPHPLC on an analytical C_18_ column (Deltapak, C_18_, 300×7.8 mm, 15 µ, 300 Å) using acetonitrile-water as eluent, respective peaks (P3E–P9E) were collected and assayed for antiplasmodial potency against *P.falciparum* 3D7. Pools (P6 and P9) were rechromatographed to collect three sub-fractions from each peak (P6Ea–P6Ec, P9Ea–P6Ec). These six sub-fractions were assayed for antiplasmodial potency against 3D7 and Dd2 strains of *P.falciparum*. Figure 1 summarizes the schematic extraction and reverse phase fractionation of extract of Harshringar leaves.

### In vitro cultivation of *P. falciparum*

CQ-sensitive (3D7) and CQ-resistant (Dd2) strains of *P.falciparum* were used *in vitro* blood stage culture to test the antiplasmodial efficacy of Harshringar leaves extract and its RPHPLC fractions. The culture was maintained at the Malaria Research Laboratory, International Centre for Genetic Engineering and Biotechnology (ICGEB), New Delhi, India. *P.falciparum* culture was maintained according to the method described by Trager and Jensen [22] with minor modifications. *P.falciparum* 3D7 and Dd2 cultures were maintained in fresh O^+^ve human erythrocytes suspended at 4% hematocrit in RPMI1640 (Sigma) containing 0.2% sodium bicarbonate, 0.5% Albumax, 45 µg/L hypoxanthine, and 50 µg/L gentamicin and incubated at 37°C under a gas mixture 5% O_2_, 5% CO_2_, and 90% N_2_. After every 24 hrs, infected erythrocytes were transferred into fresh complete medium to propagate the culture.

### Drug dilution

Chloroquine was from Sigma. Stock solution of CQ was prepared in water (milli-Q grade) and test compounds were in DMSO. All stocks were diluted with culture medium to achieve the required concentrations (in all cases except CQ, the final solution contained 0.4% DMSO, which was found to be non-toxic to the parasite). Drugs and test compounds were then placed in 96-well flat bottom tissue culture grade plates (Corning).

### Assay for antiplasmodial activity

Crude extracts and purified fractions were evaluated for their antiplasmodial potency against *P.falciparum strains* 3D7 and Dd2. For drug screening, SYBR green I-based fluorescence assay was setup as described [23]. Sorbitol synchronized parasites were incubated under normal culture conditions at 2% hematocrit and 1% parasitemia in the absence or presence of increasing concentrations of plant extracts. CQ was used as positive control, while 0.4% DMSO was used as the negative control. After 48 hrs of incubation, 100 µl of SYBR Green I solution {0.2 µl of 10,000 X SYBR Green I (Invitrogen)/mL} in lysis buffer {Tris (20 mM; pH 7.5), EDTA (5 mM), saponin (0.008%; w/v), and Triton X-100 (0.08%; v/v)} was added to each well and mixed twice gently with multi-channel pipette and incubated in dark at 37°C for 1 h. Fluorescence was measured with a Victor fluorescence multi-well plate reader (Perkin Elmer) with excitation and emission wavelength bands centered at 485 and 530 nm, respectively. The fluorescence counts were plotted against the drug concentration and the 50% inhibitory concentration (IC_50_) was determined by analysis of dose-response curves.

### UV-Visible Spectroscopy

Spectral studies were carried by spectrophotometer (Perkin-Elmer). All solvents used for spectral studies were of analytical grade. 3 mL quartz cuvettes were used for all studies. 110 µg/mL, 90 µg/mL, 180 µg/mL, 80 µg/mL and 40 µg/mL of 40%, 60%, 80%, 100% MF and pool of 60%–80% MF respectively were used for spectral studies against methanol as blank.

## Results and Discussion

Harshringar holds a reputed position as a medicinal plant in different systems of medicine in India including Ayurveda. The juice of its leaves in different forms has been advocated for acute, chronic as well as intermittent fever [12]. However antiplasmodial activity guided fractionation of the leaves extract of this plant has not yet been reported. In the present report antiplasmodial potencies of Harshringar leaves extract and its RPHPLC purified fractions have been studied against chloroquine sensitive (*Pf*3D7) and chloroquine resistant (*Pf*Dd2) strains of blood stage *P.falciparum* in red blood cell culture.

Crude ethanolic extract (CEE) of Harshringar leaves showed only modest potency (IC_50_ 77±7 µg/mL) against *P.falciparum* 3D7. We surmised that this modest potency may be related to the fact that crude plant extracts contain a number of complex molecules, only a few of which may be active. In such a scenario, the resultant activity of crude extract may increase/decrease/abolish depending on synergistic/antagonistic interactions among/between various types of complex molecules present in extract [24]. Badam et al., [19] reported the minimal efficacy dose (MED_50_) of ethanol extract of Harshringar leaves against CQ sensitive strain of *P.falciparum* in the range of 1000–1200 µg/mL. Mishra et al., [20] reported inhibition of *P.falciparum* by the 50% ethanolic extract of Harshringar leaves both *in vivo* and *in vitro*. Even as the crude extract (IC_50_ 77±7 µg/mL) was found to be largely inactive, we subjected it to activity guided RPHPLC fractionation in the hope of finding some molecules which may be present in trace amounts and yet be quite potent.

### Reverse phase chromatography of CEE

**Figure 2:**
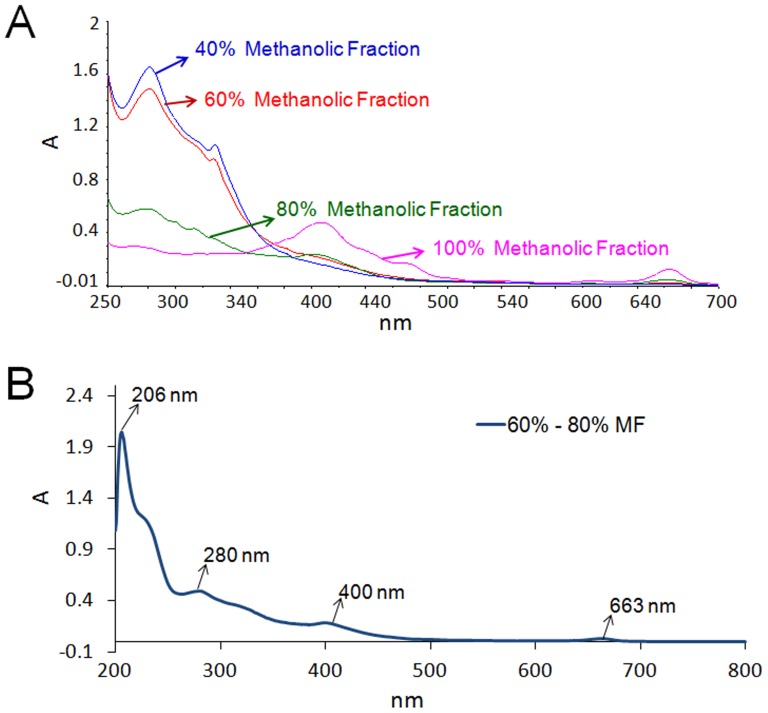
UV-visible spectra of fractions of Harshringar leaves extract. (A) Spectra of 40%, 60%, 80%, 100% MF were recorded against methanol as blank. 110 µg/mL, 90 µg/mL, 180 µg/mL, 80 µg/mL of 40%, 60%, 80%, 100% MF respectively were used for spectral studies. (B) UV-visible spectra of pool of 60%–80% MF (40 µg/mL).

**Figure 3:**
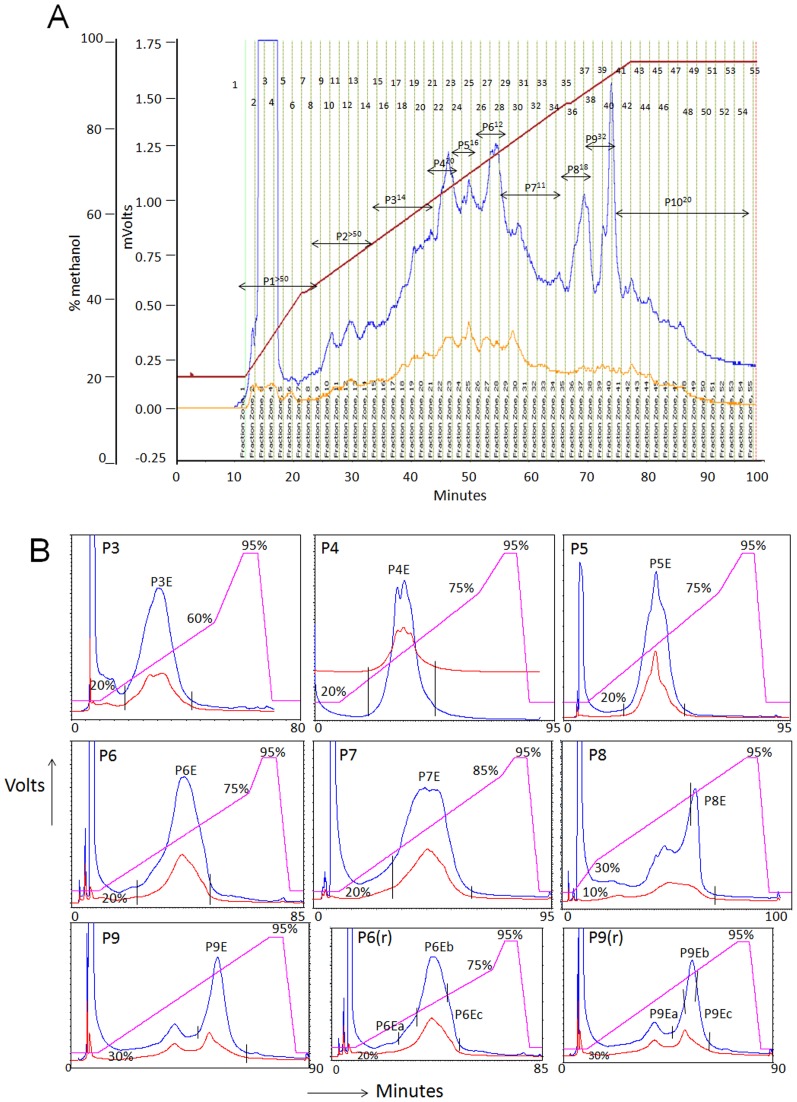
2^nd^ and 3^rd^ dimension RPHPLC fractionation of Harshringar Leaves extract. Panel A shows semi-prep RPHPLC of 60%–80% MF of Harshringar leaves. 55 fractions (indicated as numbers against fraction bars) were collected, judiciously pooled as indicated by horizontal lines to obtain 10 pools (P1–P10). A_214_ (blue line) and A_280_ (brown line). Superscripts represent IC_50_ (µg/mL) values against *P.falciparum* 3D7. Panel B shows 3^rd^ dimension RPHPLC chromatograms of pools P3–P9 of Harshringar leaves. All chromatograms were acquired at 214 nm (blue line)/280 nm (red line) except P6 and P9 which were acquired at 214 nm (blue line)/254 nm (red line). ∼2 mg/200 µL of each of the pools (P3–P9) was injected onto an analytical reverse phase C_18_ column (Deltapak, C_18_, 300×7.8 mm, 15 µ, 300 Å) using acetonitrile-water linear gradient (pink line with % values indicating % acetonitrile) at a flow rate of 2 mL/min. Peaks were collected as shown by vertical lines and denoted as P3E–P9E (Table 1). P6 and P9 were rechromatographed ({P6 (r) and P9 (r)} to collect each peak into three slices of sub fractions (P6Ea–P6Ec, P9Ea–P9Eb). Peaks and peak fractions were assayed for antiplasmodial potency against *P.falciparum*.

Glass column reverse phase chromatography of CEE was used to fractionate the crude extract. The yields obtained were 14.6% (Water Fraction), 5% (20% MF), 2.5% (40% MF), 3.5% (60% MF), 6% (80% MF), 24% (100% MF), 0.6% (50% ACNF), 0.2% (75% ACNF) of the extract loaded on column. However among the nine fractions, only two fractions 60% MF (IC_50_ 14.5±0.7 µg/mL) and 80% MF (IC_50_ 13±4.2 µg/mL) displayed promising antiplasmodial potency (Table 1). The two active fractions were pooled together and subjected to further RPHPLC fractionation. UV-Visible spectra of the pooled fractions showed strong absorbance in the range of 200–300 nm (Figure 2). Dual wavelength detection (214/254 nm or 214/280 nm) was used during RPHPLC. Based on chromatogram (Figure 3 A), 55 fractions obtained during semi-prep RPHPLC were judiciously pooled together to obtain pools P1–P10.

**Table 1:** Antiplasmodial potencies of Reverse phase chromatographic fractions of Harshringar leaves extract.

Fractions (1^st^ Dimension RPCC)	IC_50_ (µg/mL) *Pf* 3D7	Pools (2^nd^ (Dimension RPHPLC)	IC_50_ (µg/mL) *Pf* 3D7	Fractions (3^rd^ Dimension RPHPLC)	Antiplasmodial potency IC_50_ (µg/mL)	
					*Pf* 3D7	*Pf* Dd2
Water Fraction (WF)	>100^*^	P1	>50^*^	P1E (ND)^c^	(ND)^c^	(ND)^c^
20%Methanol fraction (MF)	>100^*^	P2	>50^*^	P2E (ND)^c^	(ND)^c^	(ND)^c^
40% MF	>50<100^*^	P3	13.75±8.8^***^	P3E (18–41 min^b^)	12.5^***^	(ND)^c^
60% MF^a^	14.5±0.7^***^	P4	19.75±1.0^**^	P4E (22–47 min^b^)	>25^**^	(ND)^c^
80% MF^a^	13.0±4.2^***^	P5	15.75±5.3^**^	P5E (25–51 min^b^)	23^**^	(ND)^c^
100% MF	39.0±9.9^**^	P6	12±2.8^***^	P6E (25–51 min^b^)	18^**^	(ND)^c^
50% ACN fraction (ACNF)	66.5±12^*^			P6Ea (25–35.5 min^b^)	38^**^	*7.5±2.1* ^***^
75% ACNF	20^**^			P6Eb (35.5–46 min^b^)	*6±2.8* ^***^	*7.5±3.6* ^***^
Isopronol fraction (IPF)	>100^*^			P6Ec (46–51 min^b^)	*6±1.4* ^***^	*7±1.4* ^***^
		P7	11.25±1.7^***^	P7E (31–62 min^b^)	22^**^	(ND)^c^
		P8	17.5±2.1^**^	P8E (57–67 min^b^)	>25^**^	(ND)^c^
		P9	32^**^	P9E (47–62 min^b^)	22.5^**^	(ND)^c^
				P9Ea (47–52.5 min^b^)	11±1.4^***^	14.5±2.1^***^
				P9Eb (52.5–58 min^b^)	39±2.8^**^	29.5±7.8^**^
				P9Ec (58–62 min^b^)	38.5±5^**^	39±4.2^**^
		P10	20^**^	P10E (ND)^c^	(ND)^c^	(ND)^c^

Data shown are expressed as the mean of triplicates ± SD. Potencies for active fractions (IC_50_<8 µg/mL) are shown in italics.

a Fractions which were combined together,

b Time boundaries given correspond to the vertical lines shown in each chromatogram of Fig. 3B,

c not done.

ACN: Acetonitrile, RPCC- Reverse phase glass column chromatography. CQ (100 nM, 1000 nM) were used as positive controls for *Pf*3D7 and *Pf*Dd2 respectively. For details of chromatographic separations, see Materials and methods and Figures 1 and 3. Symbols used for varying antiplasmodial potency are *** (Promising Activity, IC_50_: 5–15 µg/mL), ** (Moderate Activity, IC_50_: >15–50 µg/mL), and * (Inactive, IC_50_: >50 µg/mL).

According to WHO recommendations and previous works [25]–[27] anti-plasmodial activities of plant extracts have been classified as follows: highly active extracts with IC_50_<5 µg/mL, promising activity at 5–15 µg/mL, moderate activity at 15–50 µg/mL and inactive at >50 µg/mL. Out of the ten fractions tested, P3 (IC_50_ 13.75±8.8 µg/mL), P6 (IC_50_ 12±2.8 µg/mL) and P7 (IC_50_ 11.25±1.8 µg/mL) showed promising activity; P4 (IC_50_ 19.75±1.1 µg/mL), P5 (IC_50_ 15.75±5.3 µg/mL), P8 (IC_50_ 17.5±2.1 µg/mL), P9 (IC_50_ 32 µg/mL) and P10 (IC_50_ 20 µg/mL) showed moderate activity, while P1 (IC_50_>50 µg/mL) and P2 (IC_50_>50 µg/mL) were considered inactive (Table 1).

Progressive 2^nd^ and 3^rd^ dimensional RPHPLC purification led from marginal to significant increase in potency in some instances while a significant decline in potency was observed in other cases. Thus the 2^nd^ dimensional antiplasmodial IC_50_ (µg/mL) values of P3 (13.7) and P9 (32) dropped to 12.5 and 22.5 respectively after 3^rd^ dimensional separation. However in contrast, the 2^nd^ dimensional potencies of P4–P8 showed a significant decrease in potency following the 3^rd^ dimensional chromatography (Table 1). One of the hallmarks of activity guided purification is the trend of increasing potency with increasing purity. This trend appears to be reflected in the cases of P3 and P9. However decrease in potency with purification may suggest association of drug like activity with not necessarily a single molecule but rather with an intermolecularly interacting complex of two or more molecules. If chromatography results in disruption of such complexes, it can result in decreased potency with increased purity. In our studies, this seems to be the case with P4–P8. The possibilities of such molecular synergy and its loss have been proposed earlier [28].

P6 and P9 were rechromatographed with the aim of finding if different portions of the broad peaks may be associated with different potencies against *P. falciparum*. As shown (Figure 3 B), the front, the middle and the tail ends of each of the two peaks were collected separately. Interestingly the IC_50_ value for each of these subfractions indicated that some of them were far more active than the others. Thus the IC_50_ *Pf* 3D7 (µg/mL) associated with the subfractions of P6 were 38, 6 and 6. Similarly the IC_50_ (µg/mL) associated with the subfractions of P9 were 11, 39 and 39 respectively (Figure 3 B, Table 1). It is interesting to note that P6Ea (*Pf* 3D7 IC_50_ 38 µg/mL) showed an IC_50_ 7.5 µg/mL against the CQ resistant *Pf* Dd2 (Table 1).

Despite systematic efforts we were not able to identify any single molecule as the active principle of Harshringar leaves, responsible for antiplasmodial potency. Nevertheless the work described here is expected to lead to further efforts to identify the active antiplasmodial principle(s) of *Nyctanthes arbor-tristis* and to elucidate their exact mechanism of action.

### Hypothesis about possible compounds of *Nyctanthes arbor tristis* leaves which may exhibit antiplasmodial activity

The antiplasmodial activity in fractions could possibly have its origin in the alkaloids, phytosterols, phenolic compounds and iridoid glycosides that are found in *Nyctanthes arbor tristis* leaves. However the most probable phytoconstituents that may be responsible for the observed antiplasmodial activity may belong to the class of iridoid glycosides found in *Nyctanthes arbor tristis* leaves. This class of molecules has been reported to inhibit trypanothione reductase (TryR), a validated drug target enzyme of the Leishmania parasite [29]. TryR is a member of pyridine nucleotide-disulfide oxidoreductase family whose other members e.g. Thioredoxin reductase (TrxR) and Glutathione Reductase (GR) are found in human malaria parasite *P.falciparum* [30]. We surmise that iridoid glycosides present in the Harshringar leaves may target one or more oxidoreductases present in *P.falciparum*. Our hypothesis is consistent with the fact that inhibitors of Trypanothione Reductase are known to inhibit the growth of *P. falciparum* in culture [31].

### Conclusion

Here we have for the first time fractionated the Harshringar leaf extract and located antiplasmodial activity in specific fractions. The results achieved with this work constitute a proof that Harshringar is a promising plant with regard to anti-malarial phytotherapy and support continuous investigation of this plant to discover new anti-malarials.
